# System mapping to strengthen youth crisis response: findings from a multi-county California pilot

**DOI:** 10.3389/fpubh.2025.1704401

**Published:** 2026-01-21

**Authors:** Ashley Long, Robert D. Blagg

**Affiliations:** Agile Visual Analytics Lab (AVAL), Luskin School of Public Affairs, University of California, Los Angeles, Los Angeles, CA, United States

**Keywords:** system mapping, community systems, suicide prevention, crisis response, injury surveillance

## Abstract

Youth suicide is a pressing public health concern requiring coordinated, cross-sector responses. This research presents findings from a pilot initiative involving ten California counties that used a multi-site participatory system mapping design to assess and strengthen youth crisis reporting and response systems. Funded through the Children and Youth Behavioral Health Initiative and supported by the California Department of Public Health, the pilot used visual tools to identify gaps, clarify roles, and promote shared understanding across sectors. System maps focused on six key components of crisis systems, supporting communities in developing targeted action plans and promoting system accountability. Results demonstrated that the mapping process catalyzed improvements in follow-up care, school and hospital coordination, and postvention practices. Challenges included navigating structural differences among partners and sustaining engagement. Findings suggest that system mapping is a replicable and equity-centered approach to the development of public health infrastructure.

## 1 Introduction

Youth suicide is a complex and multifaceted public health issue that demands coordinated, cross-sector responses. Communities often struggle to understand roles, responsibilities, and intervention pathways during crises, while fragmented systems and inconsistent protocols delay timely care. These breakdowns in coordination contribute to suicide-related morbidity and mortality and reflect deeper systemic challenges (1). A systems approach to suicide prevention may address these gaps by providing infrastructure that supports real-time communication, equitable care access, and data-informed responses across agencies (2, 3).

To address these challenges, the Youth Suicide Reporting and Response Pilot, funded through the Children and Youth Behavioral Health Initiative (CYBHI), supported ten counties in strengthening their youth crisis systems. With guidance from the California Department of Public Health (CDPH) and a technical assistance provider, counties engaged in system mapping to assess infrastructure, identify service gaps, and guide improvements. Pilot funding ran from January 2023 through June 2025 and primarily focused on youth ages 0–25 years old and their caregivers.

Understanding the complex interplay of relationships, institutions, and contextual dynamics within communities is essential for effective intervention. System mapping enables stakeholders to visualize these interconnected elements, offering insights into how individual and collective behaviors are shaped (4, 5). Making systems visible helps clarify roles, surface gaps, and foster more strategic and collaborative solutions to social challenges (6, 7).

System mapping significantly enhances public health practice by providing a holistic framework to address systemic barriers and improve cross-sector collaboration. Unlike traditional linear approaches, it emphasizes feedback loops, relationships, and emergent properties that are core principles of systems thinking (5, 8). This approach has proven valuable in diverse public health domains, from chronic disease prevention to mental health promotion (3). Recent work by van den Akker et al. (3) and Giabbanelli et al. (13) also shows how systems mapping can uncover data gaps and improve surveillance infrastructure in youth suicide prevention.

California's *Striving for Zero* initiative similarly emphasizes the importance of building statewide coordination, standardizing prevention efforts, and improving system visibility to reduce suicide across age groups (9). This paper examines both the outcomes of system mapping across participating counties and evaluates system mapping as a strategic tool for improving youth suicide prevention infrastructure. We aim to document key implementation changes and assess the utility of participatory system mapping methods as a replicable approach for strengthening public health infrastructure.

## 2 Methods

### 2.1 System mapping design

System mapping is a structured, visual method that helps stakeholders collectively depict the services, actors, and policies within a given system. In this pilot, the process was informed by principles of systems thinking and participatory evaluation (4, 5).

Implementation of the pilot looked very different across the 10 counties. Pilot leads included public health departments, behavioral health departments, and one county office of education. All counties received extensive technical assistance (TA) to develop their work plans and build local capacity. These variations presented a need for tools that the TA could utilize to help counties better understand their existing ecosystems. Building off of California's previous efforts through Striving for Zero (9), the TA team (comprised of experts within the field of youth suicide prevention) developed a self-assessment tool that they completed with pilot counties to existing suicide prevention, reporting and response strategies. A self-assessment was only the first step, though, as communities then needed to be able to visually see their responses and develop action steps.

The system maps themselves had two primary goals of visualizing: (1) how a youth at risk of suicide or who has a suicide attempt navigates through the youth crisis reporting and response system, and (2) what consistently happens within a community when there is a youth suicide.

#### 2.1.1 Key components

To be able to have clear components of what the map should include, a series of meetings were conducted between the TA providers, CDPH, and the evaluation team. These meetings focused on identifying the needed system components, clarifying the language used for the items within each component, and understanding how labels might apply across different communities. It was determined that the map templates would contain 6 primary components.

- *Prevention and outreach*: what types of prevention activities and trainings exist in the community? Does the community have a strategic plan or a youth advisory board?
- *Entry points and rapid reporting*: how do youth and their families/caregivers enter the crisis continuum system and receive ongoing support? What is the notification process? How does this vary for minors and adults?
- *Immediate response*: what processes are utilized to determine appropriate post-ventions?
- *Available youth/family supports*: after a suicidal crisis, what are the typical responses and resources provided?
- *Follow-up (continuity of care)*: when and how are any follow-up services (one month or more later) conducted to determine the success of the response or the need for additional services? Are there community systems in place for tracking youth suicide trends?
- *System policies and supports*: what supports and policies are in place across different community settings to help transition youth and families/caregivers through the system?

**Figure 1:**
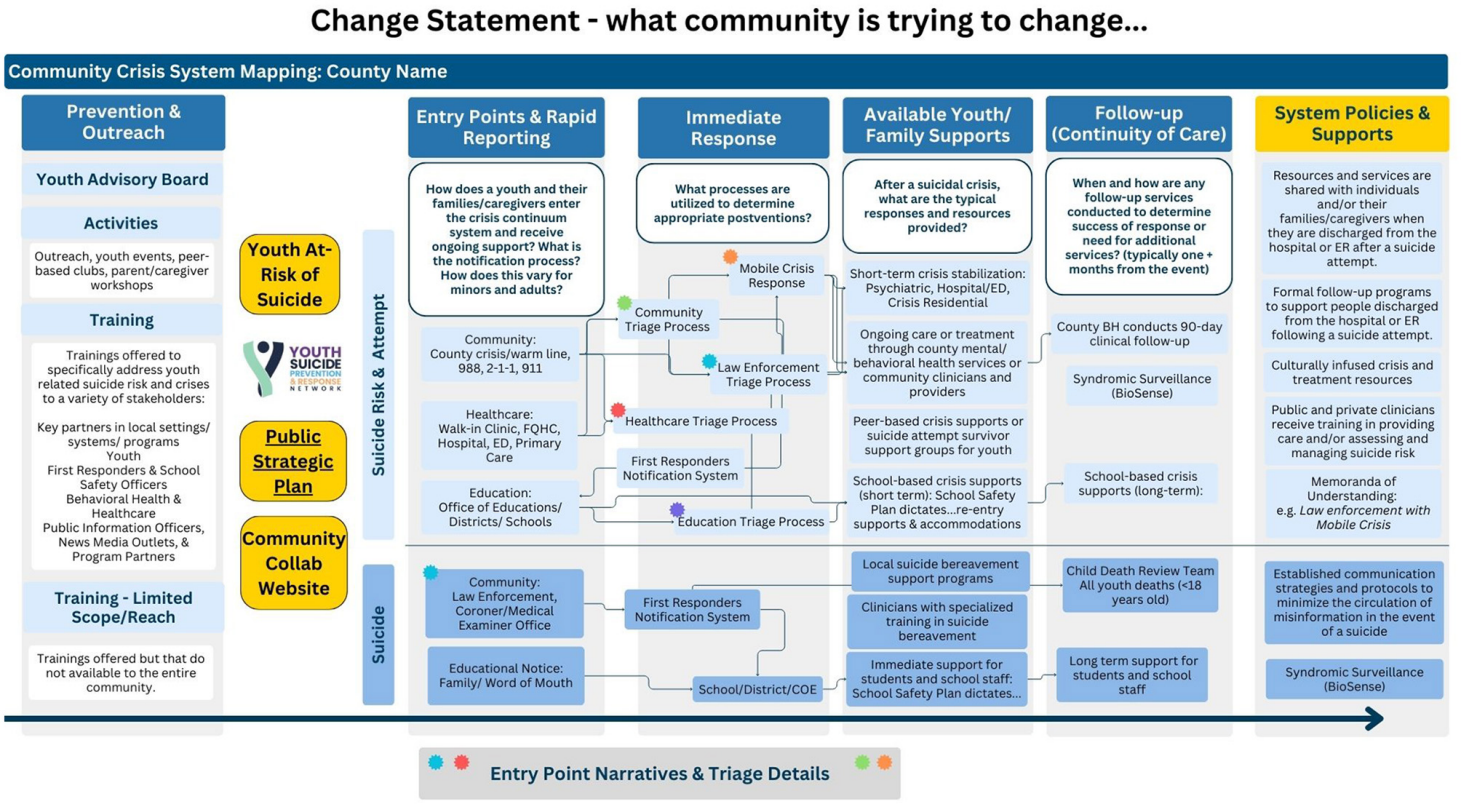
Example system map.

Beyond these components, counties focused not just on the boxes on the visual map, but time was spent analyzing response times and triage/notification processes across the different entities represented on the map, highlighting the significance of the lines, not just the entities/systems (Figure 1).

### 2.2 System mapping process

Counties followed a six-step mapping process, beginning with self-assessment and culminating in the creation of initial system maps and local change statements. Each county met with its assigned TA provider to complete its self-assessment. The sessions lasted about 60–90 min and counties completed a template with questions around the primary component areas. Open-ended fields were included throughout each section to allow TA providers to also gather other helpful, qualitative information about the county's systems.

**Figure 2:**
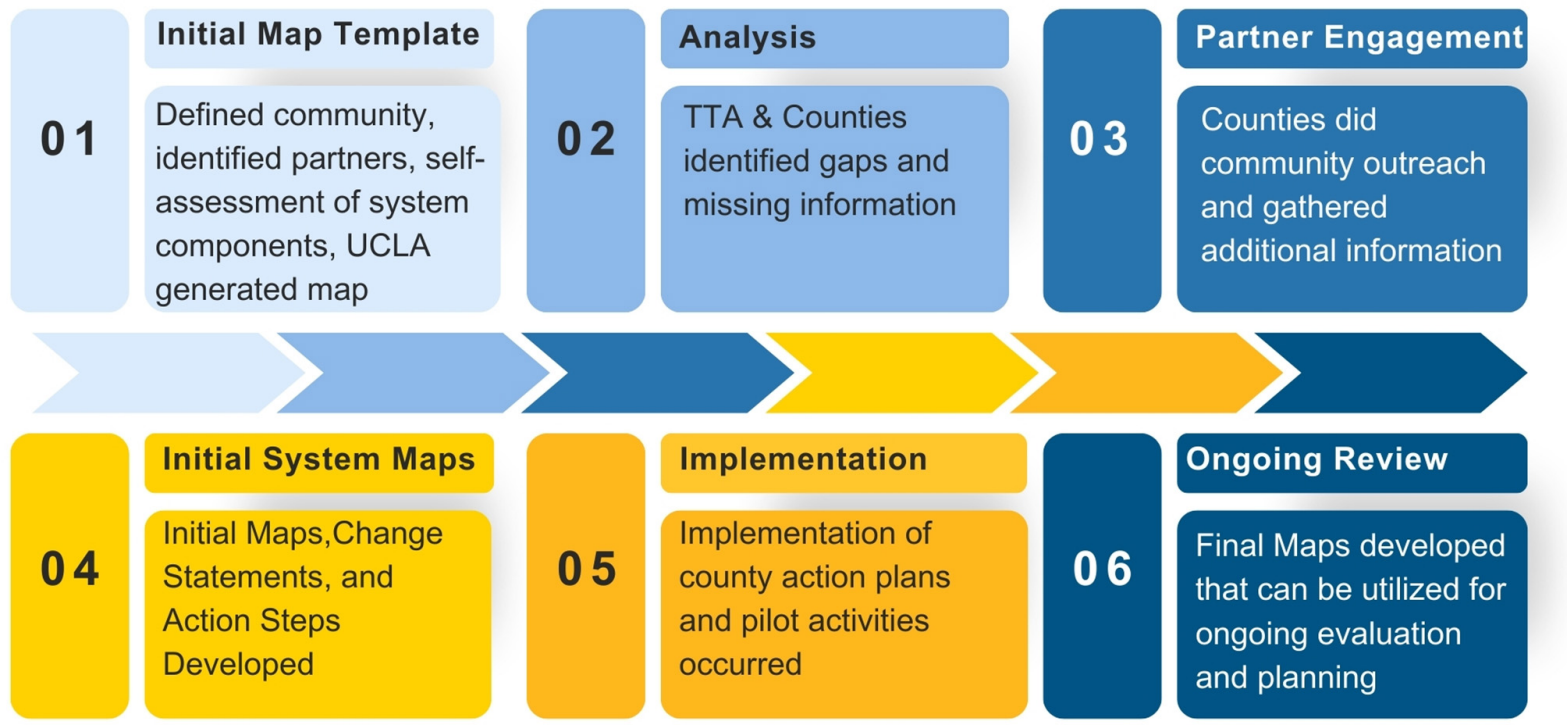
System mapping process.

Evaluation and TA teams created pre-filled map templates based on self-assessment data, which counties then refined through facilitated community discussions. The TA providers were able to virtually introduce the template system maps and then attend community meetings to help counties analyze their systems. The system maps were displayed throughout the process and in many instances even printed into posters for discussion (Figure 2).

Utilizing the initial system mapping template, the TA Team worked closely with county teams from early 2024 through April 2025 to create and refine comprehensive flow maps of their youth suicide crisis response systems. These maps detailed key entry points, triage procedures, response times, available supports, continuity of care services, system policies, and prevention efforts for youth at risk of suicide, those who have attempted suicide, or community and family members in the event of a suicide death. The TA Team provided intensive, hands-on support, including facilitating partner meetings, revising map content, and assisting counties in gathering and interpreting critical documentation, such as school suicide prevention plans and training policies. They also developed and analyzed surveys to better understand school-based responses and protocols, particularly with educational partners.

Throughout the process, counties worked closely with a variety of stakeholders, including but not limited to county public health, county behavioral health, hospitals/emergency departments, mobile crisis units, community service providers, public schools, and law enforcement.

### 2.3 Analysis

Across the pilot, System Flow Maps were produced for each county, at the baseline (June 2024) and a final map (March 2025). These artifacts captured the strengths, gaps, and opportunities within each county's crisis response system. Through ongoing collaboration, the TA Team guided counties in analyzing and interpreting their local system structures, surfacing successful practices, identifying service gaps, and developing actionable next steps. In addition to this technical support, the TA Team created opportunities for counties to build their capacity in describing and presenting their systems through local and cross-county share-out sessions.

The evaluation team conducted directed content analysis (10) of county materials, including baseline (June 2024) and final (March 2025) System Flow Maps. We treated map nodes (actors/services), edges (hand-offs/data flows), and attributes (protocols, time standards, eligibility, training) as units of analysis. Using a structured differencing checklist, we recorded Additions, Removals, Refinements, Timing changes, and policy/protocol adoptions for each county and synthesized these into county-level change statements. We then used a cross-county matrix analysis (11) to identify recurring system-change levers, domains where multiple counties showed movement (e.g., crisis coordination, school integration, continuity of care, postvention), and reporting counts (out of 10) alongside examples. This process yielded specific, documentable points of system development during the pilot while avoiding unsupported causal claims.

## 3 Results

The visual maps were pivotal for engagement: they helped stakeholders identify service gaps, clarify roles, and prioritize action. As one TA provider noted, ‘Having the map displayed during the conversation created discussion between different partners. They quickly determined that they were missing important pieces of information and set an action item to survey all the schools...' Some counties described that one or two people within their community held details about what their system looked like. The completion of the system map allowed that information to be displayed and will allow for future accessibility in sharing the system details, as well as prepare for eventual staffing and leadership changes.

The Analysis and Partner Engagement steps of the process became essential levers of change within the pilot; many communities advanced their systems in ways that would not have been possible without the system mapping. Consistent with findings in other participatory mapping initiatives (3, 6), counties reported that system mapping catalyzed collaborative problem-solving and enhanced interagency communication. The process also further supported evidence that community and system factors are an essential component when planning system initiatives (8).

A main learning from the system mapping process was the value of the process itself and the provided technical assistance to facilitate learning. Through the process, several positive outcomes occurred:

- **Action planning:** the system mapping process provided a platform for community action planning. The structured tools equipped counties with visuals to guide conversations and set direction for both action steps during the pilot and beyond.
- **Collaboration and new partnerships:** as gaps were identified within county systems, the system mapping process led to a natural exploration of different system partners. Sometimes these partnerships were renewed, and other times they were completely new. Several counties reported establishing collaborative relationships as a result of building their system maps and working together to meet community needs.
- **Gap identification:** building on the themes above, some counties were able to use the map to advocate for supports that were identified as missing. Beyond missing supports, one county was also able to identify equity disparities within their mapping process itself, acknowledging that key community representation was missing from their conversations.

Findings also showed that between July 2024 and April 2025, counties demonstrated notable progress in enhancing youth suicide prevention systems across eight key domains. Training and prevention initiatives expanded, with widespread implementation of evidence-based programs such as QPR (Question, Persuade, Refer), ASIST (Applied Suicide Intervention Skills Training), CAMS (Collaborative Assessment and Management of Suicidality), and Youth Mental Health First Aid. Youth engagement deepened through advisory boards and peer-led efforts, while cultural responsiveness improved through targeted outreach (for example one county developed a cultural responsiveness training and another designed outreach to tribes in their region). Crisis response systems became more coordinated, with clearer triage protocols and the introduction of youth-specific models like ORCA (Outreach, Referral, Care, and Access) and IHART (Intensive Home and Resilience Team). Schools improved their integration with mental health systems by standardizing protocols and strengthening collaboration with county agencies, while healthcare systems made strides in screening and discharge planning, despite limited integration with primary care settings.

Continuity of care efforts advanced through standardized follow-up timelines, expansion of post-hospitalization teams, and increased use of peer and family supports. Postvention strategies were strengthened with more school-based plans, LOSS Teams, and improved outreach to survivors, though youth-specific bereavement services remain limited. All counties completed system flow maps and developed or updated strategic plans, reinforcing partnerships across sectors. Data collection and use also improved, with more counties contributing to CalVDRS and syndromic surveillance systems, launching dashboards, and enhancing data-sharing across hospitals and EMS networks (Table 1).

**Table 1:** Findings table.

**Theme area**	**Common practices**	**Emerging innovations/expansions**	**Remaining gaps or needs**
Training and prevention	Most counties offer standardized trainings for school staff, parents, and providers, including suicide prevention basics and mental health awareness.	Train-the-trainer programs, cultural responsiveness modules, and expansion to non-traditional partners (e.g., libraries, youth councils).	Inconsistent coverage across sectors; limited reach to tribal communities, smaller districts, or out-of-school settings.
Crisis response access & coordination	All counties are integrating national and local helplines with mobile response teams and developing clearer triage protocols.	Some counties provide 24/7 mobile response with clear response times and online directories; several are piloting alternatives to police response.	Need for standardized public messaging and clarification between access points (e.g., helplines vs mobile teams).
School system integration	Most counties are engaging school districts in mapping and reviewing suicide prevention, intervention, and re-entry plans.	Development of countywide models for school-based response and postvention; use of structured checklists and training supports.	Gaps in postvention procedures and formal communication protocols following a suicide-related event.
Healthcare system integration	Many counties have outlined hospital discharge planning and follow-up protocols; some use standardized screening tools in emergency settings.	Peer navigation teams and follow-up services linked to emergency departments and behavioral health programs.	Limited behavioral health coordination with some hospitals; challenges with data-sharing and continuity after discharge.
Follow-up and continuity of care	Most counties aim for follow-up within 72 hours of a crisis, with referrals to local services and case management.	Peer-led models, family engagement strategies, and extended follow-up (up to 3 months) introduced in several areas.	Follow-up less consistent for youth not admitted to hospitals or not engaged in ongoing care.
Postvention and bereavement support	Many counties are beginning to map postvention practices and provide basic grief support resources.	Peer-led grief groups, immediate outreach teams, and school-focused postvention protocols developed in several counties.	Few counties have formal notification systems or long-term supports for survivors of suicide loss.
System coordination and strategic planning	All counties used system flow mapping to assess service gaps and promote cross-sector collaboration.	Some counties now hold regular interagency planning meetings or have integrated suicide prevention into countywide plans.	Ongoing need for sustaining cross-sector collaboration beyond grant timelines and ensuring youth and family voice in planning.
Data and surveillance	Most counties participate in state or local surveillance (e.g., syndromic data or crisis response logs).	Some have dashboards, suicide fatality review teams, or “data-to-action” alerts shared with schools and providers.	Limited real-time data sharing; disparities in hospital participation; underuse of data in decision-making.

### 3.1 Limitations

Systems are inherently complex, and navigating their intricacies presents persistent challenges across counties. Among the most frequently identified issues was a disconnection between system components, manifesting in several ways. Counties noted that information about school processes was often limited, structures between Behavioral Health and Public Health systems differed substantially, and crisis response times varied by setting, making comparisons difficult. More support and time for structured data collection may provide more valid and comparable system maps.

In addition to these structural challenges, many counties reported difficulties with the coordination process itself. Engaging multiple stakeholders required significant time and effort, and obtaining buy-in from non-funded partners proved particularly challenging. These barriers often forced counties to recognize existing gaps in their understanding of the broader system. This was an advantage of the process, but caused several counties to adjust their approaches, narrowing their scope to focus on more targeted activities than originally planned. More time and support for building stakeholder buy-in may prove to maintain a more complete scope for the system mapping process.

A further, cross-cutting challenge involved the need for shared terminology and language. To ensure that all participants interpreted system maps consistently, counties often began their processes with introductions and clarifications of key terms. This issue became even more pronounced when comparing efforts across counties, as terminology and descriptions were not standardized statewide. Collectively, these findings underscore the importance of developing common definitions within and across systems to enhance communication, facilitate collaboration, and support shared learning across jurisdictions.

## 4 Discussion

System mapping provided counties with a structured yet adaptable method for examining and improving youth crisis response systems. Although participants described the process as labor-intensive, they emphasized its essential role in making complex systems both visible and actionable. The participatory nature of the approach fostered a sense of ownership and collaboration across sectors, while the resulting visual representations served as dynamic reference points to guide ongoing system improvement efforts.

Several key lessons emerged from the implementation of this approach. First, visualization proved to be a powerful catalyst for dialogue. Making abstract and interconnected processes visible enabled stakeholders to engage in more timely and productive discussions about system complexity and interdependence. This finding aligns with other participatory frameworks that employ mapping to promote shared understanding among diverse participants [e.g., Heinze et al. (12)].

Second, the system mapping process demonstrated the value of balancing flexibility with standardization. While each county's map was shaped by local context and priorities, shared structural components provided a coherent foundation that supported cross-system comparability. This combination of local adaptability and a standardized core offers a potential model for collaborative work across multiple jurisdictions or communities.

Third, sustained engagement required both time and dedicated support. Counties that were most successful invested heavily in building relationships, often extending participation beyond funded partners to include a broader range of stakeholders. Technical assistance (TA) providers played a critical role in translating the mapping tool, fostering understanding, and maintaining momentum throughout the process.

Finally, the maps themselves were most effective when treated as living tools rather than static products. Counties that continued to revise and update their maps in response to new data and dialogue experienced greater progress in system planning and alignment. This iterative use of system maps highlights their potential not only as diagnostic instruments but also as evolving frameworks for continuous learning and collaboration.

The system mapping process contributed to concrete system improvements across the pilot. The visual representation of service pathways and responsibilities helped counties identify and correct surveillance gaps—for instance, by encouraging integration with CalVDRS, establishing syndromic surveillance protocols, and launching new dashboards for real-time alerts. In continuity of care, mapping revealed inconsistencies in follow-up timelines, prompting the creation of post-hospitalization teams, clearer handoffs, and peer-led follow-up strategies, particularly for youth discharged from emergency departments. In the area of postvention, the process exposed missing communication protocols and helped drive the adoption of LOSS teams, school-based postvention plans, and outreach efforts for suicide loss survivors. These examples illustrate how making systems visible was instrumental to building infrastructure and addressing persistent fragmentation.

System mapping represents a practical, participatory tool for improving service coordination in complex community systems. For health promotion professionals, it offers a replicable framework for assessing and improving multisectoral collaboration and a platform for community action planning rooted in shared understanding. It can also serve as a mechanism to visualize change and communicate progress to diverse audiences. As with other tools for practice, success depends on facilitation, local ownership, and alignment with broader public health infrastructure (2, 3).

State agencies and philanthropic funders should also consider system mapping as a tool for building local capacity, especially when supporting cross-sector public health work. Investments in visualization tools and TA can foster shared accountability and align interventions across jurisdictions. The tool also provides a platform to create shared language and protocols to improve inter-county learning and collaboration. Furthermore, the system mapping framework provides a vehicle for sustaining community systems. Counties can better demonstrate gaps in their system, and visualizations can inform strategic planning and community engagement while also providing a tool that can be revisited on an ongoing basis.

## 5 Conclusion

The System Flow Mapping process proved to be both a powerful tool and a transformative experience for pilot counties. Through structured phases and intensive technical assistance, counties developed deep insight into the pathways youth follow during suicide-related crises. The process enabled communities to visualize how youth interact with complex systems—from schools and healthcare settings to community resources—and to identify opportunities for improved coordination, communication, and culturally responsive care.

Across all ten counties, the mapping process catalyzed new partnerships, exposed longstanding gaps, and reinforced strengths. Through the development of Baseline and Final Maps, Change Statements, and Summary Statements, counties clarified their system structures and committed to meaningful change. It provided a shared framework for measuring progress and identifying the steps needed to strengthen continuity of care, postvention services, and system accountability.

Looking ahead, the system mapping tool offers substantial potential for ongoing use and adaptation. Several counties are exploring setting-specific applications (e.g., mapping only the education sector), regional adaptations for large counties, and simplified versions for public use. These simplified maps could be featured on county websites to help youth and families navigate available resources. Additionally, counties are planning to use system maps to support grant proposals, establish or expand suicide prevention councils, and maintain collaborative momentum with cross-sector stakeholders. Lessons learned during the pilot emphasize the importance of minimizing jargon, ensuring shared language, and including direct links to services to make maps accessible and actionable for broader audiences.

Sustained implementation and refinement of these maps, alongside investment in infrastructure, cross-sector data sharing, and training will be critical for long-term impact. As communities across the nation grapple with fragmented systems of care, especially in behavioral health and youth services, system mapping emerges as a replicable tool to support health equity, local ownership, and actionable change. External TA support plays an important role in helping communities with system mapping efforts maintain accountability in moving work forward and engage cross-sector partners over the long development period needed to create system change. Continued investment in the use and refinement of such frameworks can help institutionalize systems thinking in public health and ensure that community voices remain central to reform efforts.

## Data Availability

The datasets presented in this article are not readily available because the included data sets belong to the California Department of Public Health and participating counties in California. Data was not summarized into any public report. Requests to access the datasets should be directed to Ashley Long, amlong@ucla.edu.
